# Reduction aortoplasty supported by pericardial patch, early results and outcome at Queen Alia Heart Institute

**DOI:** 10.1186/1749-8090-8-S1-O8

**Published:** 2013-09-11

**Authors:** H Altaani, A Jaber

**Affiliations:** 1 Cardiac Surgery Department, Queen Alia Heart Institute, Amman, Jordan

## Background

Reduction aortoplasty is an alternative method of treatment for ascending aorta aneurysm without involvement of the aortic root especially in high risk patients to decrease the cross clamp time, our study is to evaluate the early results and outcome of longitudinal reduction aortoplasty supported by pericardial patch at our institute.

## Methods

From 2006 till end of 2011, 37 patients with aneurysm of the ascending aorta without involvement of the aortic root underwent longitudinal reduction aortoplasty supported by pericardial patch which was sewed to the lateral wall of the ascending aorta by 5/0 prolene. The average age was 70.9 year ±4.2, females were 28 (75.7%), the average ejection fraction was 42.1%. The size of the ascending aorta was less than 55 mm in all patients; all patients underwent other cardiac procedure at the same time. 33 patients (89%) aortic valve replacement, 22 patients (59.5%) aortic valve replacement with CABG, 4 patients (11%) CABG alone, 7 patients (19%) were redo aortic valve replacement. Mean follow-up was 2 year.

## Results

Preoperative mortality was in 2 cases 5.4%, both because of heart failure because of low ejection fraction preoperatively. One year survival was 91.9%. Reoperation done in 3 cases one because of hematoma collection beneath the pericardial patch after 3 weeks of the primary operation, the other 2 was because of valve complications. No redilataion seen in any patient till the time we did this study.

## Conclusion

Reduction aortoplasty is safe alternative to the standard replacement of the ascending aorta for patients without involvement of the aortic root with mild to moderate dilatation, can be done either supported with pericardial patch or unsupported, we think that supported aortoplasty may decrease the risk of redilataion and the risk for reoperation, our early results are encouraging.

